# SIRT3 is required for liver regeneration but not for the beneficial effect of nicotinamide riboside

**DOI:** 10.1172/jci.insight.147193

**Published:** 2021-04-08

**Authors:** Sarmistha Mukherjee, James Mo, Lauren M. Paolella, Caroline E. Perry, Jade Toth, Mindy M. Hugo, Qingwei Chu, Qiang Tong, Karthikeyani Chellappa, Joseph A. Baur

**Affiliations:** 1 Department of Physiology and Institute for Diabetes, Obesity and Metabolism, Perelman School of Medicine, University of Pennsylvania, Philadelphia, Pennsylvania, USA.; 2 Children’s Nutrition Research Center, Baylor College of Medicine, Houston, Texas, USA.

**Keywords:** Hepatology, Metabolism, Fatty acid oxidation, Mitochondria, Molecular pathology

## Abstract

Liver regeneration is critical to survival after traumatic injuries, exposure to hepatotoxins, or surgical interventions, yet the underlying signaling and metabolic pathways remain unclear. In this study, we show that hepatocyte-specific loss of the mitochondrial deacetylase SIRT3 drastically impairs regeneration and worsens mitochondrial function after partial hepatectomy. Sirtuins, including SIRT3, require NAD as a cosubstrate. We previously showed that the NAD precursor nicotinamide riboside (NR) promotes liver regeneration, but whether this involves sirtuins has not been tested. Here, we show that despite their NAD dependence and critical roles in regeneration, neither SIRT3 nor its nuclear counterpart SIRT1 is required for NR to enhance liver regeneration. NR improves mitochondrial respiration in regenerating WT or mutant livers and rapidly increases oxygen consumption and glucose output in cultured hepatocytes. Our data support a direct enhancement of mitochondrial redox metabolism as the mechanism mediating improved liver regeneration after NAD supplementation and exclude signaling via SIRT1 and SIRT3. Therefore, we provide the first evidence to our knowledge for an essential role for a mitochondrial sirtuin during liver regeneration and insight into the beneficial effects of NR.

## Graphical abstract

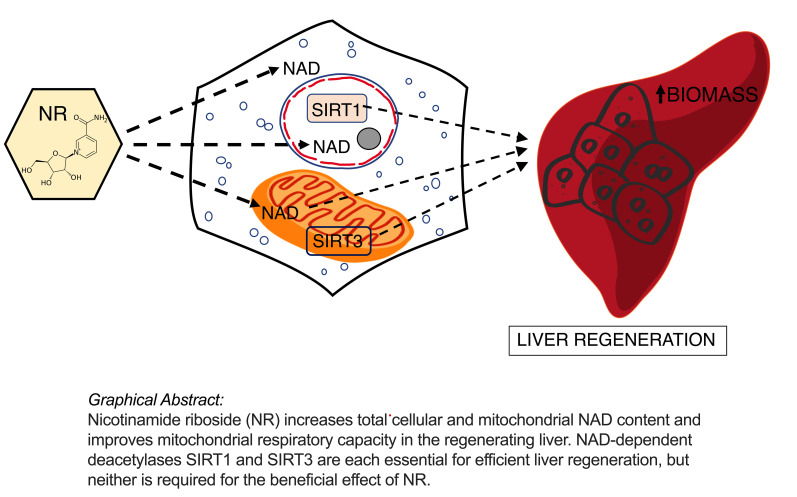

## Introduction

Hepatocytes lost owing to acute injury or over the course of chronic disease are replaced by mitotic division of the remaining mature cells. The ability to activate the acute regenerative response can be the determining factor between survival and death after a toxic or traumatic injury, transplant, or surgical resection. Within hepatocytes, the switch from a nondividing to a dividing state involves substantial remodeling of metabolism (1, 2) and is demanding in terms of both energy and the coordination of flux through multiple anabolic pathways. Moreover, synthesis of new hepatocytes must be accomplished while the remaining tissue supports multiple indispensable liver functions, including glucose production. Despite decades of study, there remains no clinically proven therapy to support regeneration in patients at risk for liver failure or following an acute injury or surgical intervention.

The early stages of liver regeneration are characterized by a transient decrease in NAD content, which has been attributed to diversion of precursors such as phosphoribosylpyrophosphate (PRPP) to nucleic acid synthesis (3–5). We recently showed that augmenting NAD synthesis, either genetically or by supplementing with the precursor nicotinamide riboside (NR), is sufficient to accelerate liver regeneration in mice (6). However, the mechanism by which NAD availability influences the rate of liver regeneration has not yet been elucidated.

In addition to its well-known role as an electron acceptor in hundreds of biochemical reactions, NAD serves as a cosubstrate for several classes of enzymes with important signaling functions (7–10). These include the sirtuins (SIRT1–7), a family of NAD-dependent deacylases that differ in their tissue distribution, subcellular localization, and enzymatic activity. The nuclear sirtuin SIRT1 has previously been shown to be required for normal liver regeneration (2, 9) (although overexpression may be harmful; ref. 11), and in other settings it has been shown to mediate key effects of NAD precursors, including promoting mitochondrial function and the clearance of fat from hepatocytes (12–15). Recently, mice lacking another nuclear sirtuin, SIRT6, in hepatocytes were also shown to exhibit delayed regeneration (16). We view SIRT6 as unlikely to be responsible for the effects of NR because it has a high affinity for NAD (*K_D_* of approximately 27 μM [ref. 17], well below reported nuclear NAD levels [ref. 18]), suggesting that it would be less likely than other sirtuins to respond to modest changes in NAD concentration. We recapitulated the requirement for SIRT1 in normal liver regeneration but found that NR remained effective in these animals, leading us to consider other mechanisms.

Although liver regeneration is an energetically demanding process that requires substantial flux through mitochondrial pathways, the role of mitochondrial sirtuins has not been investigated. Mice lacking each of the 3 mitochondrial sirtuins, SIRT3–SIRT5, are viable and unremarkable under basal conditions (19). However, SIRT3 has emerged as the major isoform responsible for deacetylation of mitochondrial proteins (19), and loss of SIRT3 renders mice susceptible to ischemic and other injuries (20–22). SIRT3 has been shown to influence the metabolic capacity of mitochondria under stresses, including in hepatocytes (23–25), and is responsible for the ability of NR to protect against hearing loss in mice (26), suggesting that it might also mediate the effect of NR on liver regeneration. Here, we demonstrate that mice lacking SIRT3 in hepatocytes have major defects in liver regeneration but are surprisingly still fully responsive to NR. Therefore, we show for the first time to our knowledge that a mitochondrial sirtuin is essential for normal liver regeneration and demonstrate that NR remains effective in the absence of either SIRT1 or SIRT3, the major sirtuin isoforms responsible for deacetylation in the nucleus and mitochondria, respectively.

## Results

### The beneficial effects of NR in the regenerating liver are independent of SIRT1.

**Figure 1:**
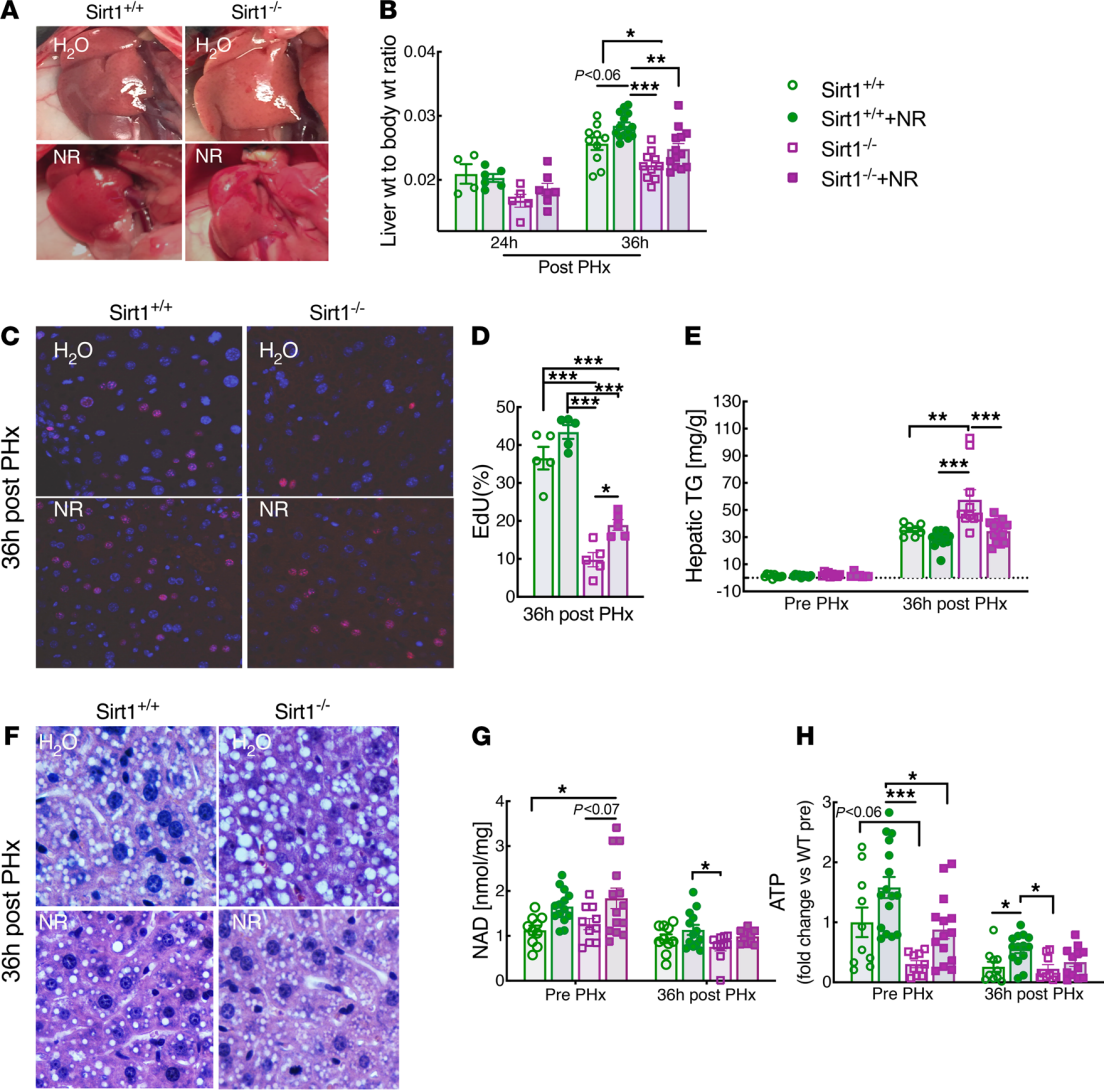
SIRT1 is not required for nicotinamide riboside to enhance liver regeneration. *Sirt1* liver-specific-KO mice (denoted as *Sirt1^–/–^*) and floxed littermates lacking Albumin Cre (denoted as *Sirt1^+/+^*) were subjected to partial hepatectomy (PHx) after 2 weeks of nicotinamide riboside (NR) supplementation. (**A**) Photographs of regenerating livers 36 hours after PHx. (**B**) Liver-to-body weight ratios at 24 hours (*n* = 4–7 per group) and 36 hours (*n* = 10–14 per group) after PHx. (**C**) Immunofluorescence showing proliferating hepatocytes (red) detected by EdU and counterstained with DAPI (blue). (**D**) Quantification of EdU-positive hepatocytes (*n* = 5 per group). (**E**) Hepatic lipid content as determined by triglyceride assay. (**F**) Representative liver sections stained with H&E. (**G** and **H**) Hepatic NAD and ATP content in PHx livers prior to and after 36 hours from control or NR-treated mice. High power field, 40× objective, 400× magnification for images in **C** and **F**. Data are shown as the mean ± SEM. *P < 0.05; **P < 0.01; ***P < 0.001; 1-way ANOVA followed by Tukey’s post hoc test (**B**, **D**, **E**, **G**, and **H**).

We previously observed that enhanced liver regeneration in NR-treated mice correlates with accelerated lipid clearance (6). The NAD-dependent deacetylase SIRT1 promotes liver regeneration and mitochondrial lipid metabolism and has been shown to mediate key effects of NAD precursor supplementation, including lipid clearance and fatty acid oxidation (FAO) in mouse models of diet-induced obesity and alcohol-induced steatosis (2, 9, 12–15, 27, 28). In fact, it has been suggested that NAD biosynthesis and SIRT1 activity are intimately coupled across many tissues (14, 29), and beneficial effects of knocking out other key NAD consumers such as PARP1 and CD38 have been attributed to sparing NAD for SIRT1 or SIRT3 (30–32). To directly test whether SIRT1 is the mechanism linking NR to enhanced liver regeneration, we supplemented NR in mice lacking SIRT1 in hepatocytes. Loss of SIRT1 (Supplemental Figure 1, A and B; supplemental material available online with this article; https://doi.org/10.1172/jci.insight.147193DS1) was sufficient to confer decreased respiratory capacity in isolated mitochondria from regenerating livers (Supplemental Figure 1, C and D). Moreover, deletion of *Sirt1* impaired liver regeneration, as evidenced by a decreased liver-to-body weight ratio, reduced indices of hepatocyte proliferation (Figure 1, A–D), and lead to hepatic steatosis as reflected in histology and hepatic triglyceride content (Figure 1, E and F). All of these effects were opposite to those of NR supplementation, supporting the model that NR might work via SIRT1. However, we found that the efficacy of NR treatment was preserved, with equal or greater incremental effects in the mice lacking hepatic SIRT1 (Figure 1, A–H). Overall, the effects of SIRT1 and NR status were additive. Thus, despite separately playing an important role, SIRT1 does not appear to be the primary mechanism by which increased NAD is able to promote liver regeneration.

### NR improves mitochondrial respiratory capacity in the regenerating liver.

**Figure 2:**
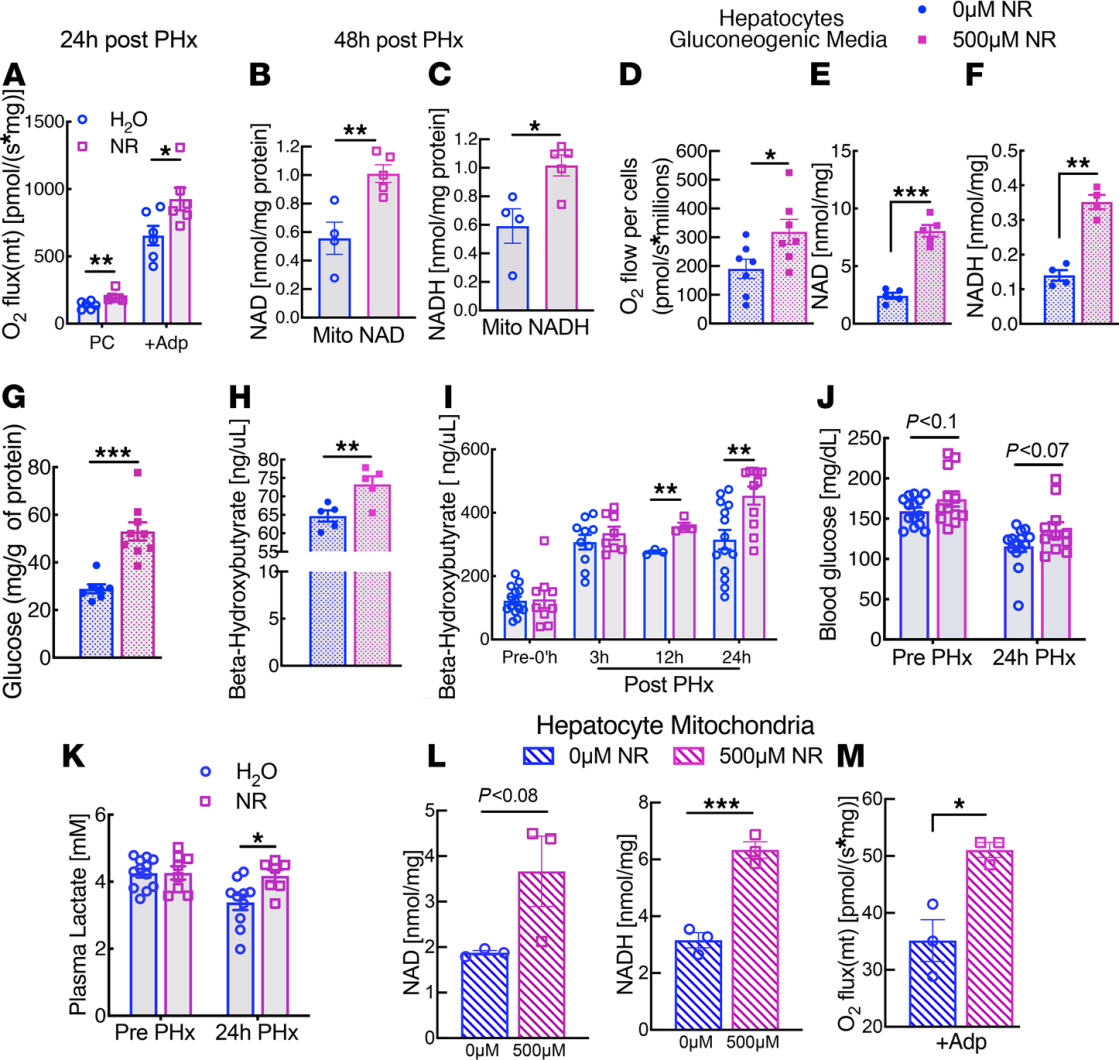
NR enhances mitochondrial respiration in regenerating liver and in primary hepatocytes. Male C57BL6/J mice 10–16 weeks of age were supplemented for 2 weeks with NR at a dose of 500 mg/kg body weight and then were subjected to two-thirds partial hepatectomy (PHx). (**A**) Mitochondria from livers of H_2_O- and NR-treated mice were isolated 24 hours after PHx (*n* = 6 per group) and were analyzed for fatty acid oxidation capacity using palmitoyl carnitine as a substrate. (**B** and **C**) Mitochondrial NAD and NADH content in organelles from control or NR-treated mice at 48 hours after PHx (*n* = 4–5 per group). Primary hepatocytes were isolated from overnight fasted C57BL6/J WT mice, cultured in glucose-free media with gluconeogenic substrates with or without NR for 5 hours. Mitochondria were then isolated from treated or untreated hepatocytes (*n* = 4–9 per group). (**D**) Oxygen consumption of primary hepatocytes. (**E** and **F**) Hepatocyte NAD and NADH content after treatment. (**G** and **H**) Final glucose and β-hydroxybutyrate concentration in the media. (**I**) Plasma β-hydroxybutyrate content at the indicated time points after PHx in H_2_O- and NR-treated mice (*n* = 3 per group at 12 hours and *n* = 8–14 per group in all other time points). (**J** and **K**) Blood glucose and plasma lactate levels in prior to and 24 hours after PHx in H_2_O- and NR-treated mice (*n* = 11–14 per group). (**L**) Mitochondrial NAD and NADH content in treated and untreated hepatocytes (*n* = 3 per group). (**M**) State 3-coupled mitochondrial oxygen consumption using pyruvate plus malate as substrates. In vivo data are represented by open circles and squares, and hepatocyte data are denoted by filled circles and squares. Data are shown as the mean ± SEM. *P < 0.05; **P < 0.01; ***P < 0.001; 2-tailed Student’s *t* test.

To understand why livers regenerate better in the presence of NR, we next examined its effects on mitochondrial function. NR supplementation during regeneration was sufficient to enhance the ability of hepatic mitochondria to oxidize fatty acids (Figure 2A). Concordantly, both NAD and NADH content of isolated mitochondria were increased after NR supplementation (Figure 2, B and C). In contrast to the results in regenerating livers, we found that NR did not affect the respiratory capacity of mitochondria isolated from uninjured livers (Supplemental Figure 2, A and B).

### NR enhances respiration and biosynthetic capacity in isolated hepatocytes.

Although there is no widely accepted cell culture model for liver regeneration, placing primary hepatocytes in glucose-free media with gluconeogenic substrates similarly places a high biosynthetic demand on the cells. We found that 5 hours of treatment with 500 μM NR was sufficient to increase overall respiratory capacity in primary hepatocytes under gluconeogenic conditions (Figure 2D), correlating with a substantial increase in NAD and NADH content (Figure 2, E and F). Our results are in contrast to those of Dall et al. who found that treating primary hepatocytes with NR at the same dose had no effect on mitochondrial NAD levels or respiration (33). One possible resolution is the effect of culture conditions, as we deliberately stressed primary hepatocytes with gluconeogenic conditions, whereas Dall and colleagues employed standard media. Consistent with the increased in mitochondrial activity, NR increased the output of both glucose and β-hydroxybutyrate in hepatocytes (Figure 2, G and H), confirming that it improves the capacity for production of circulating fuels. In vivo NR also increased plasma β-hydroxybutyrate levels at 12 or 24 hours after hepatectomy (Figure 2I). Blood glucose trended higher, and lactate concentrations were increased by NR treatment (Figure 2, J and K), consistent with improved gluconeogenesis. Given the important roles of NAD in the mitochondrial matrix, we next considered whether improvements in respiratory capacity might be mediated directly by mitochondrial NAD levels. NAD and NADH concentrations were substantially higher in mitochondria isolated from hepatocytes after 5 hours of NR treatment in culture (Figure 2L), similar to the effect of NR supplementation in vivo (Figure 2A and Supplemental Figure 2C). In addition, the isolated mitochondria retained their enhanced respiratory capacity, suggesting a rapid intrinsic change in the organelles following NR treatment (Figure 2M) rather than a change in substrate delivery or mitochondrial mass.

### SIRT3 is required for liver regeneration but does not mediate the benefits of NR.

**Figure 3:**
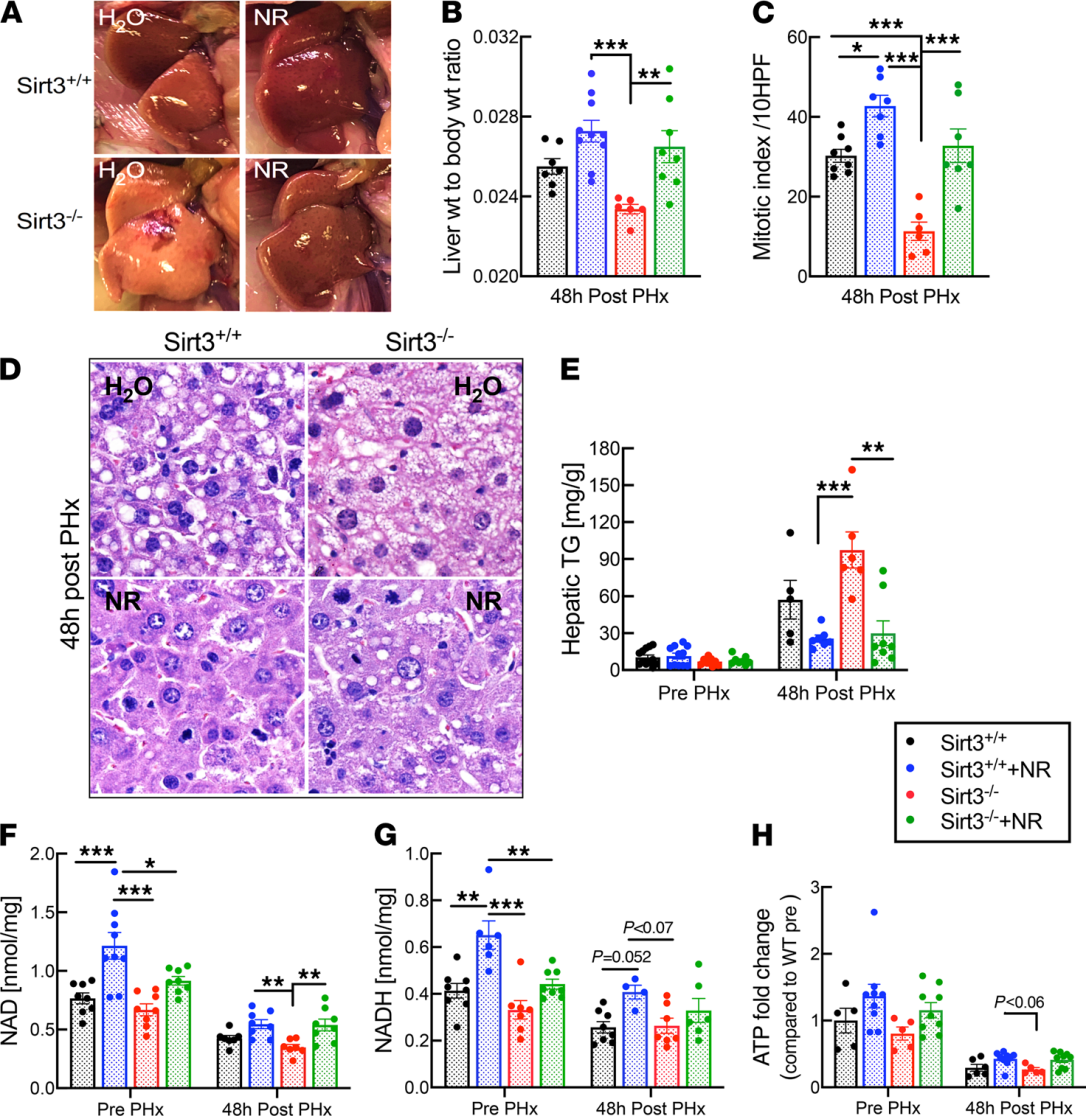
Sirt3 is essential for liver regeneration but is dispensable for the beneficial effects of NR. Liver-specific *Sirt3*-KO mice (denoted as *Sirt3^–/–^*) or floxed controls (*Sirt3^+/+^*) were subjected to two-thirds PHx and analyzed 48 hours later (*n =* 5–10 per group). (**A**) Photographs of regenerating livers. (**B**) Liver-to-body weight ratios 48 hours after PHx. (**C** and **D**) Quantitation of mitosis as determined by counting mitotic figures in hepatocytes in H&E-stained sections under high power. Representation of the images are shown in (**D**). High power field, 40× objective, 400× magnification for images in **D**. (**E**) Hepatic triglyceride content in livers from H_2_O- and NR-treated mice prior to and after PHx. (**F** and **G**) NAD and NADH content in livers from H_2_O- and NR-treated mice prior to and after PHx. (**H**) ATP content in livers from H_2_O- and NR-treated mice prior to and after PHx. Data are shown as the mean ± SEM. *P < 0.05; **P < 0.01; ***P < 0.001; 1-way ANOVA followed by Tukey’s post hoc test (**B**, **C**, and **E**–**H**).

The sirtuin SIRT3 is the major mitochondrial deacetylase and has been suggested to promote FAO in an NAD-dependent manner by deacetylating multiple enzymes within the mitochondrial matrix (25). In several cases, SIRT3 has been suggested to underlie benefits of NAD supplementation (26, 34). However, the effects of SIRT3 on in vivo liver regeneration have not previously been investigated. To study the role of SIRT3, we generated a hepatocyte-specific KO model by infecting *Sirt3*-floxed mice with adeno-associated virus encoding Cre under the control of the hepatocyte-specific TBG promoter to (Supplemental Figure 3, A and B). Like whole-body *Sirt3*-KO mice (19), the hepatocyte-specific KO mice exhibited no overt abnormalities and appeared indistinguishable from WT littermates (Supplemental Figure 3). However, these mice exhibited drastically reduced regenerative capacity following partial hepatectomy (PHx). Liver-to-body weight ratio, indices of hepatocyte proliferation, hepatic TG content, and hepatic NAD, NADH, and ATP content were all deteriorated in the Sirt3-KO mice (Figure 3 and Supplemental Figure 3). Thus, the stress of liver regeneration reveals a critical role for this mitochondrial sirtuin.

Despite the lack of this key NAD-dependent enzyme, NR supplementation improved the liver weight, hepatocyte proliferation, mitotic indices, hepatic NAD, NADH, and ATP content in *Sirt3*-KO mice (Figure 3). In addition, NR lowered hepatic triglycerides to a level similar to that observed in control mice (Figure 3, D and E). These findings establish that whereas SIRT3 is necessary to support normal liver regeneration, it is dispensable for the beneficial effects of enhanced NAD synthesis.

### SIRT3 and NR independently improve mitochondrial function.

**Figure 4:**
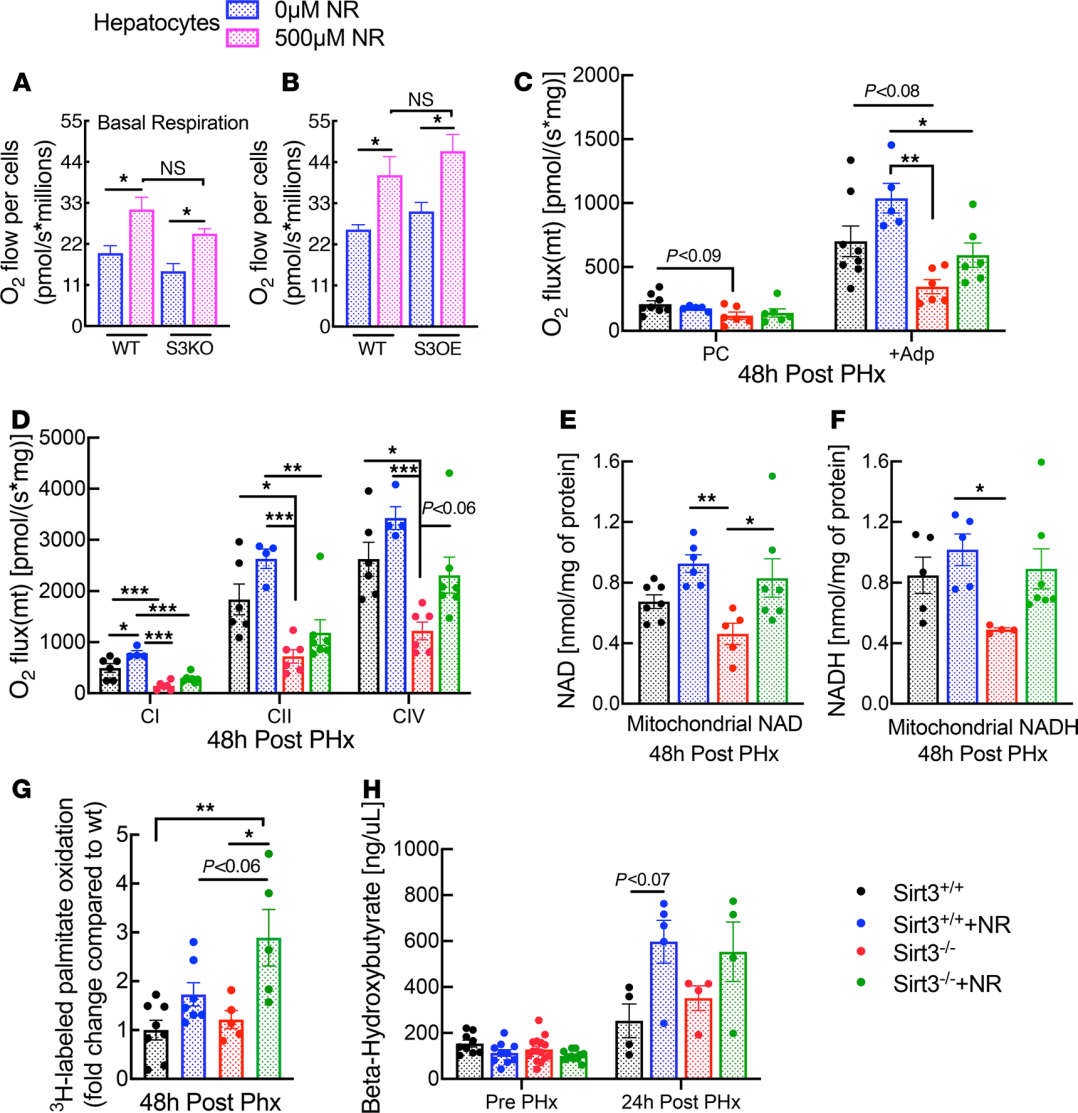
Sirt3 and NR independently improve mitochondrial function. (**A** and **B**) Primary hepatocytes were isolated from overnight fasted whole-body *Sirt3*-KO or -overexpressing mice and their respective WT littermates, cultured in glucose-free media with gluconeogenic substrates, and treated with or without NR for 5 hours before measuring oxygen consumption (*n =* 3 per group). Mitochondria were isolated from NR-treated or -untreated regenerating livers of liver-specific *Sirt3*-KO mice (*n =* 4–8 per group). (**C** and **D**) Mitochondrial respiration in organelles isolated from regenerating livers. (**E** and **F**) Mitochondrial NAD and NADH content 48 hours after PHx. (**G**) ^3^H-labeled palmitate oxidation in homogenates from livers 48 hours after PHx. (**H**) Plasma β-hydroxybutyrate concentration prior to (*n =* 9–15 per group) and 24 hours (*n =* 4 per group) after PHx. Data are shown as the mean ± SEM. *P < 0.05; **P < 0.01; ***P < 0.001; 1-way ANOVA followed by Tukey’s post hoc test (**A**–**H**).

We next tested the influence of SIRT3 expression on mitochondrial phenotypes and on the ability of NR to improve mitochondrial respiration. In hepatocytes isolated from mice lacking or overexpressing SIRT3, mitochondrial respiration trended slightly lower or higher, respectively, relative to matched controls (Figure 4, A and B). In both cases, NR increased oxygen consumption to a similar degree. Although these in vitro experiments demonstrated that NR increases hepatocyte respiration independently from SIRT3, many of the factors influencing regeneration in vivo cannot be adequately modeled in cell culture. Therefore, we next tested the influence of SIRT3 status and NR supplementation on mitochondrial respiration in vivo. The respiratory capacity of mitochondria isolated from liver-specific *Sirt3*-KO mice after PHx was consistently lower than that of littermate controls (Figure 4, C and D). Mitochondrial function was partially rescued by NR treatment such that the respiration of treated *Sirt3* KO mitochondria became similar to that of mitochondria from untreated controls. Interestingly, mitochondrial NAD and NADH levels were reduced in liver-specific *Sirt3*-KO mice, despite SIRT3 being an NAD consumer (Figure 4, E and F). NR supplementation increased mitochondrial NAD levels concurrently with improvements in respiratory capacity (Figure 4, C and D). Consistent with a SIRT3-independent mechanism of action, NR also improved FAO capacity in whole tissue lysates and increased circulating β-hydroxybutyrate levels irrespective of *Sirt3* status (Figure 4, G and H).

## Discussion

Liver regeneration is a metabolically demanding process that can determine patient survival, yet there are no therapies directed specifically toward metabolic support. We recently showed that increasing hepatic NAD content improves the regenerative capacity of the liver. This likely reflects alleviating competition for metabolic precursors that may get diverted from NAD to DNA synthesis during regeneration (3–5), but the precise mechanism by which the resulting lower NAD levels impair regeneration has not been determined. Understanding the specific mechanisms that are affected by NAD deficiency during liver regeneration has the potential to lead to new therapeutic approaches for liver injury and to provide new insight into the consequences of physiologically relevant changes in NAD concentration.

In in most of the cases in which a mechanism has been suggested for beneficial effects following NAD supplementation, it involves SIRT1 (27, 28) or in rarer cases, SIRT3 (26, 34). SIRT1 has previously been implicated in liver regeneration (2, 9) and in resolving hepatocyte lipid accumulation following NR treatment in the setting of nonalcoholic fatty liver disease (27). Moreover, it has been suggested that improved regeneration following treatment with the NAD precursor nicotinamide involves SIRT1 (35), although this was not tested with KO models. Thus, we initially set out to test whether the beneficial effects of NR in liver regeneration are dependent on SIRT1. As expected, we confirmed that the loss of SIRT1 activity in hepatocytes substantially impaired liver regeneration and delayed lipid clearance. However, NR treatment improved liver weight, hepatocyte replication, and lipid clearance even in the livers lacking SIRT1. Although *Sirt1* deletion was restricted to hepatocytes, we previously showed using genetic models that manipulation of NAD only in hepatocytes is sufficient to correspondingly enhance or worsen regeneration (6). This suggests that NAD-dependent changes in SIRT1 activity in nonhepatocyte cells of the liver are unlikely to play a key role. Thus, NR appears to improve the replicative capacity of hepatocytes in a manner that is independent of and additive with hepatic SIRT1 activity.

To gain further insight into the ability of NAD to support hepatocyte metabolism, we studied primary hepatocytes under gluconeogenic conditions. The rapid onset of increased respiratory capacity following NR treatment argues against the requirement for long-term adaptations that might result from changes in gene expression downstream of NAD-dependent signaling enzymes such as the nuclear sirtuins. However, it has been proposed that the mitochondrial sirtuin SIRT3 can influence FAO via deacetylation of multiple enzymes within the mitochondrial matrix (25), a model that is compatible with our observations. The effect of SIRT3 on liver regeneration in vivo has not previously been investigated. Therefore, we generated a hepatocyte-specific *Sirt3*-KO model and showed that loss of SIRT3 drastically impairs hepatocyte replication and promotes fat accumulation. These findings reveal an important an unrecognized role for SIRT3 in liver regeneration. However, NR treatment remained fully effective in hepatocyte-specific *Sirt3*-KO mice for all of the parameters that we assayed, indicating an independent mechanism of action.

Together, our data establish an essential role for the mitochondrial sirtuin SIRT3 in liver regeneration and provide mechanistic insight into the beneficial effects of NR. Loss of SIRT3 results in mitochondrial dysfunction, lipid accumulation, and loss of hepatocyte replication in the regenerating liver. Although increasing NAD concentration is sufficient to accelerate mitochondrial metabolism in hepatocytes or the regenerating liver, the effect is independent of both SIRT3 and its nuclear counterpart SIRT1. Although it remains formally possible that another NAD-consuming enzyme contributes to the effects of NR in vivo, our data are consistent with the view that the direct role of NAD in mitochondrial redox reactions is limiting in liver regeneration. More generally, the ability of NAD concentration to influence flux through mitochondrial pathways in this system supports the model that metabolic reactions may be directly influenced by physiologically relevant changes in NAD concentration. Thus, we identify SIRT3 as a critical new player in liver regeneration and show that NAD supplementation influences metabolism independently of both SIRT1 and SIRT3.

## Methods

### Animals, housing, and treatments.

Hepatocyte-specific *Sirt1*-KO animals were created by crossing mice containing a *Sirt1* allele with exon 4–floxed to mice expressing Cre under the control of the liver-specific albumin promoter.

WT C57BL/6 mice were obtained directly from Jackson labs at 8–10 weeks of age. Mice bearing an allele of *Sirt1* with loxP sites surrounding exon 4, which encodes the catalytic domain of the protein (36, 37), were crossed with mice expressing liver-specific Albumin Cre (JAX strain B6.Cg-Tg(Alb-cre)21Mgn/J, stock number 003574) to generate *Sirt1*^Δex4/Δex4^ animals (both parental strains on C57BL/6 background). Littermates bearing floxed alleles but lacking Albumin Cre were used as controls. *Sirt3*-floxed mice possessing loxP sites flanking exon 2–3 of the Sirtuin 3 (*Sirt3*) gene (38) were procured from Jackson labs (B6.129(Cg)-*Sirt3*^tm1.Auw^/J). For studying the role of hepatic mitochondrial FAO and steatosis, mice were injected in the retroorbital plexus with adeno-associated virus expressing either Cre recombinase (AAV-Cre) or enhanced GFP (AAV-Gfp) under the control of the thyroxine-binding globulin (Tbg) promoter, which is specific to hepatocytes. Whole-body SIRT3-deficient mice (19) and SIRT3-overexpressing mice (Baylor College of Medicine) on a C57BL/6 background were also used to generate primary hepatocyte cultures.

Partial hepatectomies were performed between the ages of 10 and 16 weeks and more than 14 days after AAV infection for animals with hepatic deletion of *Sirt3*.

In supplementation experiments, NR chloride was provided in the drinking water at 3 g/L for 2 weeks, resulting in a dose of approximately 500 mg/kg body weight based on the average water consumption per cage. NR was kept in light-protected bottles and was replaced 3 times weekly on Monday, Wednesday, and Friday.

### PHx.

Male transgenic mice (10–16 weeks old) underwent PHx according to the protocol of Mitchell and Willenbring (39, 40) between 8 am and 1 pm. Briefly, a ventral midline incision was made under isoflurane anesthesia, and the median and left lateral lobes compromising 70% of the liver were surgically removed by pedicle ligation. Animals were sacrificed at 24, 36, or 48 hours after PHx, as indicated. Mice were injected i.p. with 16 mg/kg 5-ethynyl-2’-deoxyuridine (EdU) (Molecular Probes) 5 hours before sacrifice at the 36-hour time point to capture the peak of DNA synthesis. The resected and regenerating livers were freeze-clamped in liquid nitrogen for metabolite analysis, fixed in 4% paraformaldehyde.

### Histology.

Hepatic steatosis or fatty changes were visualized, and mitotic figures were assessed in H&E-stained paraffin embedded liver sections (5 μm thickness). Mitotically active areas were first previewed under lower magnification and then counted in 10 high-power fields (40× objective, 400× magnification) for quantification of total mitotic counts. Representative photomicrographs were taken at 400× magnification using a light microscope (Olympus DP72) coupled with a digital image acquisition system.

### EdU staining.

The Click-iT EdU Alexa Fluor 594 Imaging Kit (Molecular Probes) was used to quantify EdU-positive hepatocytes in paraffin-embedded liver sections. Six random fields of each liver section containing approximately equal representation of central venous and portal triad regions were imaged and counted manually at 20× for EdU-positive cells using DAPI counterstain for normalization.

### Immunoblotting.

For Western blot analysis, freeze-clamped liver pieces were lysed using a tissue lyser (Qiagen) in RIPA buffer supplemented with Halt phosphatase inhibitors (Thermo Scientific), 1 mM nicotinamide, and 1 μM trichostatin A. Tissue lysates were probed using anti-SIRT1 (Cell Signaling, CS2028S), anti-acetylated lysine (Cell Signaling, CS9441S), anti-SIRT3 (Cell Signaling, CS5490S), anti-VDAC1 (Abcam, ab14734), and HRP-conjugated β-actin (Abcam, ab49900) Abs. Immunoreactive proteins were detected by chemiluminescence using Super Signal West Femto substrates (Pierce), and images were captured in a Bio-Rad imaging station and quantified using ImageJ (NIH).

### Isolation of primary hepatocytes.

Primary hepatocytes were isolated as previously described (41) using a modified 2-step perfusion method. The portal vein was cannulated, and the liver was perfused with liver perfusion media (Invitrogen, 17701-038) followed by liver digestion media, containing Krebs-Ringer Bicarbonate (KRB, MilliporeSigma, K-4002) supplemented with 20 mM HEPES, 500 μM CaCl_2,_ Collagenase/Elastase (Worthington, LK002066) and DNase I (Worthington, LK003170). After perfusion, the liver was removed and disrupted to release cells using cell scrapers. The cell suspension was then filtered through a 70 μm filter, centrifuged at 50*g* for 5 minutes at 4°C, washed once in Krebs-Ringer buffer, and precipitated in a 25% Percoll gradient at 120*g* for 5 minutes at 4°C. NR treatment was 500 μM, which we have found to be optimal for increasing NAD content.

### Hepatocytes culture in gluconeogenic media.

Healthy hepatocytes were plated at a concentration of 3 × 10^6^ or 1 × 10^6^ on 10- or 6-cm collagen-coated dishes, respectively, in 1 × M199 supplemented with 10% FBS, 1% Penicillin Streptomycin, Sodium bicarbonate (2.2 g/L), L-Glutamine (0.1 g/L), and 0.25% BSA.

Primary hepatocytes were cultured overnight, washed in 1 × PBS and were switched to Glucose Output Media (1× salt solution containing 118 mM NaCl, 4.7 mM KCl,1.2 mM MgSO4, and 1.2 mM KH_2_PO4 supplemented with 1.2 mM CaCl_2_, 20 mM NaHCO_3_, 20 mM HEPES, pH 7.4, and 0.025% BSA). Gluconeogenesis was then induced with glucagon and gluconeogenic substrates (20 mM lactate, 2 mM pyruvate, 10 mM glutamine, and 5 nM glucagon), with or without 500 μM NR as indicated. The cells were kept in gluconeogenic substrate media with their respective treatments for approximately 5 hours to allow accumulation of glucose in the media. The supernatant was then taken for glucose measurement by HK assay (MilliporeSigma, GAHK-20) and β-hydroxybutyrate assay (MilliporeSigma, MAK041). Cells were harvested and assessed for viability by trypan blue exclusion and immediately used for high-resolution respirometry or mitochondrial isolation or were stored at –80°C until assayed for NAD, NADH, and ATP.

### Mitochondrial isolation.

Liver tissue (approximately 100 mg) was homogenized in mitochondrial isolation buffer (MIB) (containing 210 mM mannitol, 70 mM sucrose, 1 mM EDTA, 10 mM HEPES, final pH adjusted to 7.2 using KOH and freshly supplemented with 0.25% fatty acid–free BSA), and mitochondria were isolated using differential centrifugation as previously described (42). The final mitochondrial pellet was resuspended in MIB or extracted for measurement of NAD or NADH. Protein concentration was measured by Pierce BCA Protein Assay (Thermo Fisher Scientific). For mitochondrial isolation from hepatocytes, approximately 5 × 10^6^ cells were resuspended in 1 mL MIB with 0.25% BSA and homogenized on ice using an IKA RW20 digital homogenizer at 12,000 rpm/min for 20 times. The resulting cell slurry was centrifuged at 800*g* for 5 minutes at 4°C. The supernatant was then further centrifuged at 10,000*g* for 10 minutes at 4°C. The mitochondrial fraction was collected and resuspend in MIB.

### High-resolution respirometry.

Isolated mitochondria (200 μg) from liver were resuspended in 2 mL prewarmed MIRO5 respiration buffer, containing 110 mM mannitol, 0.5 mM EGTA, 3 mM MgCl_2_, 20 mM taurine, 10 mM KH_2_PO_4_, 60 mM K lactobionate, 0.3 mM DTT, and 0.1% fatty acid–free BSA at pH 7.2. A standard substrate/inhibitor titration protocol was used for functional analysis of hepatocyte respiratory function (43). Baseline oxygen consumption was measured using high-resolution respirometry at 37°C with constant stirring (Oxygraph-2k, Oroboros Instruments). Following stabilization, real-time oxygen concentration and flux data were continuously collected using substrates and inhibitors for the ETC and FAO. CI-dependent respiration was induced by adding 10 mM pyruvate, 10 mM malate, and 1 mM ADP to the respiration chamber. To determine Cl-dependent respiration, rotenone (0.5 μM), an inhibitor of CI, was added followed by 10 mM succinate. Antimycin A (5 μM) was then added to inhibit complex III, followed by TMPD (0.5 mM) and ascorbate (2 mM) as artificial substrates for complex IV. This protocol was completed within 40 minutes. Similarly, fatty acid–dependent respiration was induced by adding 10 mM malate, 4 mM palmitoyl carnitine, and 1 mM ADP to the respiration chamber. The data were analyzed using DatLab software 4.3 (Oroboros Instruments).

### NAD metabolite extraction from liver, mitochondria, and hepatocytes.

NAD was extracted from 50 mg freeze-clamped liver, 50–100 μg isolated mitochondria, or 1 × 10^6^ primary hepatocytes in 500 μL ice-cold 0.6 M perchloric acid. Tissues were homogenized at 20 Hz for 2 minutes at 4°C using a tissue lyser (Qiagen). The insoluble materials were precipitated by centrifugation at 15,000*g* for 10 minutes at 4°C, and the clear supernatant was diluted to 1:100 in ice-cold 100 mM sodium phosphate buffer, pH 8. NAD was measured by an enzymatic cycling assay in a 96-well format modified from the procedure of Graeff and Lee (44) as previously described. Briefly, 5 μL NAD standards or diluted tissue extracts were freshly prepared and added to 95 μL cycling mix consisting of 2% ethanol, 100 μL/mL alcohol dehydrogenase, 10 μL/mL diaphorase, 20 mM resazurin, 10 mM flavin mononucleotide, 10 mM nicotinamide, and 0.1% BSA in 100 mM phosphate buffer, pH 8. Alcohol dehydrogenase reduces NAD to NADH, which in the presence of diaphorase, quantitatively reduces resazurin to the fluorescent molecule resorufin with regeneration of NAD. The cycling reaction was incubated for 30 minutes at room temperature, and the concentration of NAD was determined based on the rate of resorufin accumulation as measured by fluorescence with excitation at 544 nm and emission at 590 nm.

NADH was similarly extracted from freeze-clamped liver tissue, mitochondria, and hepatocytes in a prechilled extraction buffer (25 mM NH_4_Ac, 25 mM NaOH, 50% [v/v] acetonitrile) bubbled with nitrogen gas. Alkaline extracts were incubated at 55°C for 10 minutes to degrade any residual NAD, cooled. and centrifuged. The supernatants were diluted 1:50 in ice-cold 100 mM phosphate buffer, pH 8, for NADH measurement by cycling assay. Mitochondria and hepatocytes were separately vortexed vigorously for 1 minute and then subjected to centrifugation for 10 minutes (15,000*g*, 4°C), the clear supernatant was removed and either diluted to 1:10 or 1:5 in ice-cold 100 mM sodium phosphate buffer, pH 8, for NAD and NADH measurement.

### ATP determination.

ATP was measured in neutralized PCA extracts from either 50 mg snap-frozen liver tissue or primary hepatocytes (1 × 10^6^ cells) using an ATP determination Kit (Life Technologies) according to the manufacturer’s protocol.

### Hepatic triglyceride assay.

A total of 50 mg snap-frozen liver tissue was extracted in cell lysis buffer (140 mM NaCl, 50 mM Tris, pH 7.4, 0.1% Triton-X) and was centrifuged for 15 minutes at 20,817 x g before determination of triglycerides using the Infinity Triglyceride kit (Thermo Scientific), with glycerol as standard according to the manufacturer’s protocol.

### Plasma analysis.

Plasma triglycerides were determined using the Infinity Triglyceride kit with glycerol as a standard. Plasma nonesterified fatty acid were determined using the Wako NEFA kit with oleic acid as standard. Plasma β-hydroxybutyrate was determined using MAK041 (MilliporeSigma) and plasma L-Lactate was measured using a kit from Cayman Chemicals according to the manufacturer’s protocols. Glucose and lactate values were measured using tail vein blood with a ReliOn glucometer or Lactate PLUS meter.

*Measurement of* β*-oxidation of fatty acids from liver lysate*. A total of 500 μg protein suspension of liver homogenate was mixed with 1 mL Krebs-Ringer bicarbonate buffer containing fatty acid–free bovine serum albumin (100 mg/mL), 2.5 mM palmitic acid, 10 mM carnitine, and 4 μCi 9,10-^3^H-palmitoyl-CoA (45). The master mix was incubated in the dark at 37°C for 2 hours in a shaker, followed by Folch-based separation of 9,10-^3^H-palmitoyl-CoA and ^3^H_2_O. The aqueous phase was taken for protein precipitation by the addition of 10% tricarboxylic acid followed by centrifugation at 8000*g* for 10 minutes at room temperature. The remaining radioactive palmitoyl-CoA was eliminated by passing through a strong anion-exchange chromatography with activated AG 1-X8 chloride resin. The effluent containing the ^3^H_2_O was collected and used for scintillation counting. Each experiment was coupled with a background measurement, a sample containing no protein or liver homogenate, and was subtracted from each measurement corresponding to analysis samples.

### Statistics.

All data are shown as mean ± SEM. Comparisons between 2 groups were analyzed using unpaired or paired 2-tailed Student’s *t* test. Comparisons between 3 or more groups were analyzed using 1-way ANOVA followed by Tukey’s post hoc testing in Prism 8 (GraphPad). A *P* value of less than 0.05 was considered significant, with additional thresholds defined in the figure legends.

### Study approval.

All animal work was performed in accordance with the guidelines and with approval of the University of Pennsylvania IACUC.

## Author contributions

SM and JAB conceived the experiments. SM, JM, LMP, JT, MH, QC, CEP, and KC performed the experiments. QT generated the SIRT3-overexpressing mice. SM and JAB analyzed the data and wrote the manuscript. All authors reviewed and edited the manuscript.

## Supplementary Material

Supplemental data

## Version 1 03/09/2021

In-Press Preview

## Version 2 04/08/2021

Electronic publication

## Version 3 12/09/2021

Qiang Tong's name was spelled incorrectly
