# Supplementary material for: SIRT3 is required for liver regeneration but not for the beneficial effect of nicotinamide riboside
Source: JCI Insight. 2021 Apr 8;6(7):e147193. doi: 10.1172/jci.insight.147193 (PMC8119200; doi:10.1172/jci.insight.147193)
Supplement: Supplemental data [file jciinsight-6-147193-s005.pdf]

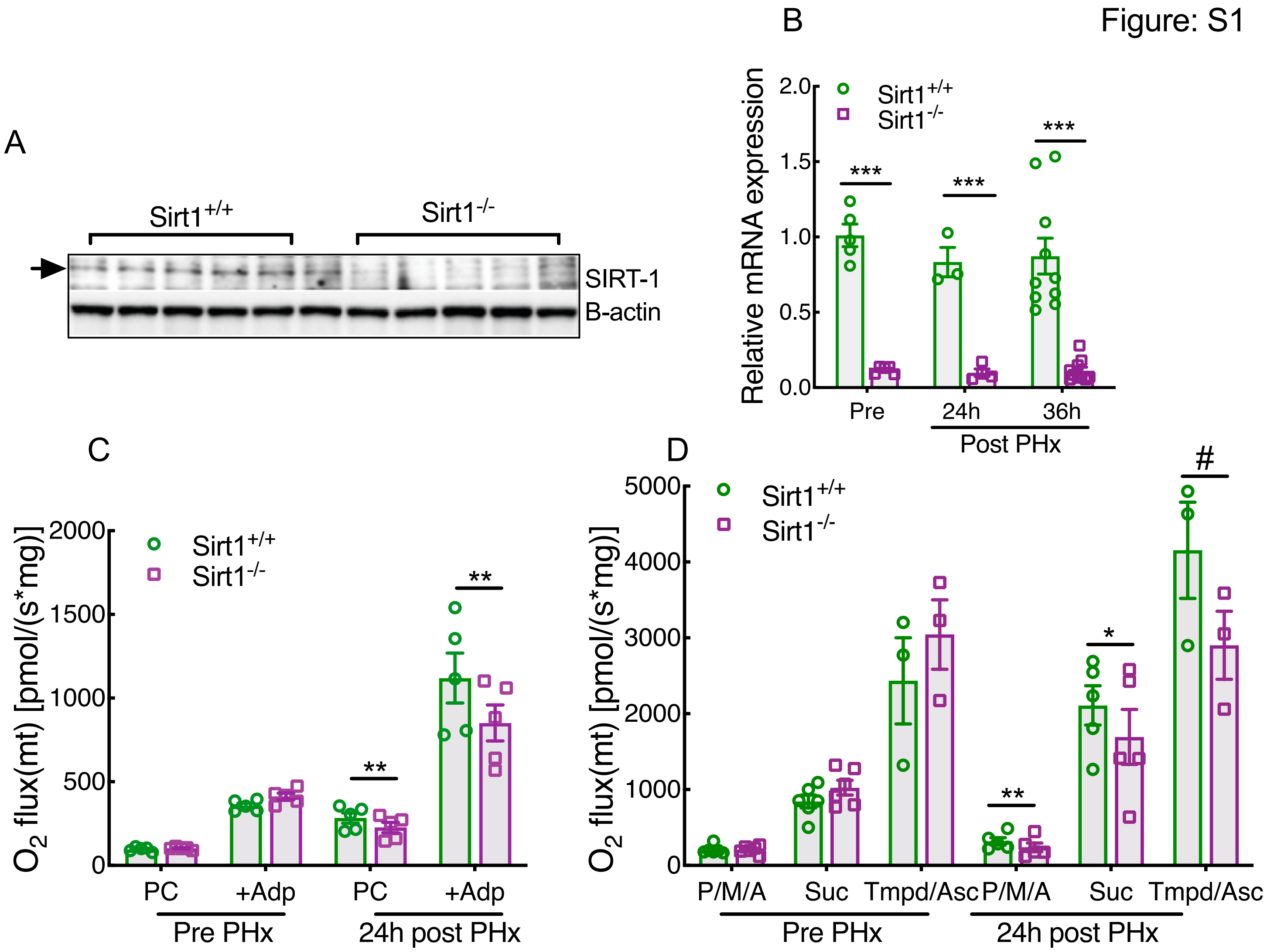

Figure S1: *Loss of Sirt1 confers a defect in mitochondrial respiration*.
Mitochondria were isolated from resected and regenerated livers of wild type and *Sirt1* knock out mice and then were subjected to state 3 coupled respiration (n=3-6 per group for 24h time point and n=10 per group for 36h post Phx).
(A, B) SIRT1 protein and mRNA expression in liver. (C, D) Oxygen consumption in isolated mitochondria using FAO (malate, palmitoylcarnitine and Adp) and non-lipid substrates (pyruvate, malate and Adp, P/M/A) in wild type and *Sirt1* knock out mice.
Error bars represent S.E.M. *, $p < 0.05$; **, $p < 0.01$; ***, $p < 0.001$.

Figure: S2

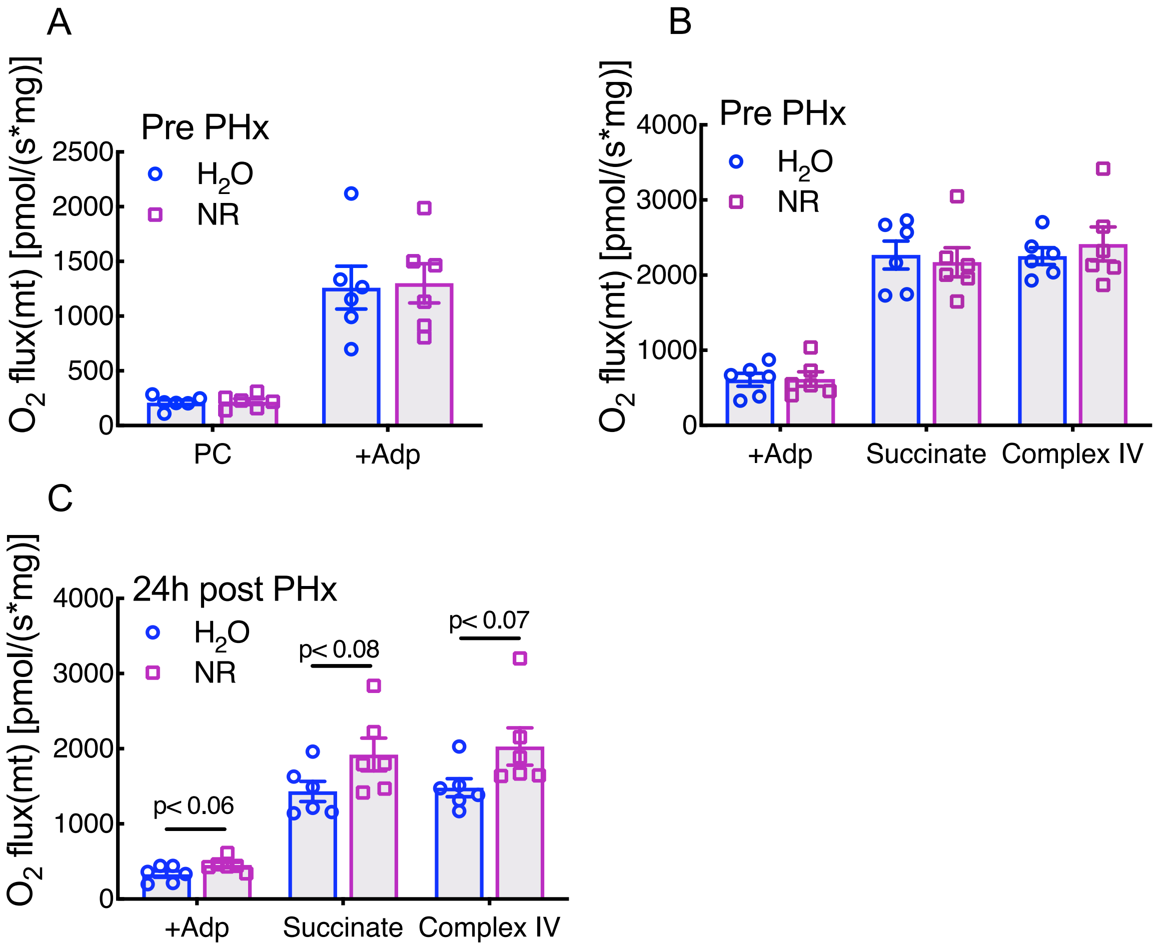

Figure S2: *Mitochondrial respiratory capacity in uninjured and 24h post PHx livers*.
(A, B) Resected liver state three coupled mitochondrial oxygen consumption using fatty acid oxidation and non-lipid substrates (n=6 per group). (C) State three coupled mitochondrial oxygen consumption using pyruvate plus malate as substrates in pre and 24h post PHx (n=6 per group).
Error bars represent S.E.M. *, $p < 0.05$; **, $p < 0.01$; ***, $p < 0.001$.

Figure: S3

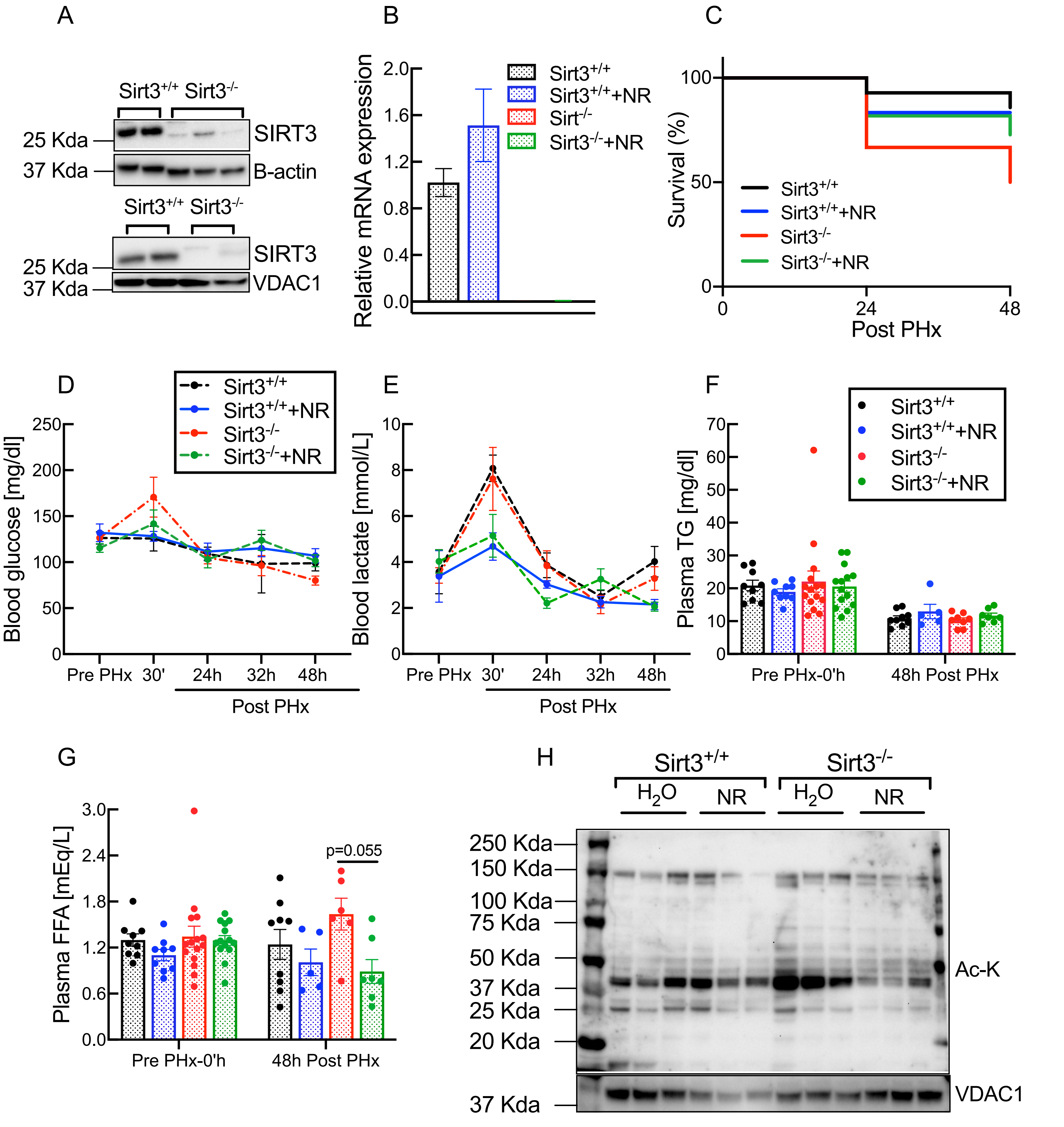

Figure S3. *Phenotypic characterization of Sirt3 null mice after partial hepatectomy.*
(A) SIRT3 protein expression in liver lysates and mitochondrial fractions. (B) *Sirt3* mRNA expression in liver. (C) *Sirt3* mice survival curve post PHx. (D, E) Blood glucose and blood lactate measured at different time points in pre and post PHx. (F, G) Plasma TG and FFA at different time points in pre and post PHx.
(H) Acetylated lysine expression in mitochondrial fractions from regenerated livers.
Error bars represent S.E.M. *, $p < 0.05$; **, $p < 0.01$; ***, $p < 0.001$ (n=4-15 per group).
